# Salivary Cortisol and Total Antioxidant Capacity (TAC) as Biomarkers of Stress in Dental Medicine Students—A Pilot Study

**DOI:** 10.3390/medicina60121972

**Published:** 2024-11-30

**Authors:** Otilia Bolos, Vanessa Bolchis, Ramona Dumitrescu, Vlad Tiberiu Alexa, Berivan Laura Rebeca Buzatu, Anca Marcu, Catalin Marian, Paula Diana Ciordas, Daniela Jumanca, Atena Galuscan, Octavia Balean

**Affiliations:** 1 Department of Dento-Facial Aesthetics, Faculty of Dental Medicine, University of Medicine and Pharmacy “Victor Babes”, 300041 Timisoara, Romania; bolos.otilia@umft.ro; 2 Translational and Experimental Clinical Research Centre in Oral Health, Department of Preventive, Community Dentistry and Oral Health, University of Medicine and Pharmacy “Victor Babes”, 300040 Timisoara, Romania; dumitrescu.ramona@umft.ro (R.D.); vlad.alexa@umft.ro (V.T.A.); berivan.haj-abdo@umft.ro (B.L.R.B.); jumanca.daniela@umft.ro (D.J.); galuscan.atena@umft.ro (A.G.); balean.octavia@umft.ro (O.B.); 3 Clinic of Preventive, Community Dentistry and Oral Health, Department I, University of Medicine and Pharmacy “Victor Babes”, Eftimie Murgu Sq. No 2, 300041 Timisoara, Romania; 4 Department of Biochemistry and Pharmacology, University of Medicine and Pharmacy “Victor Babes”, PtaEfimie Murgu Nr. 2, 300041 Timisoara, Romania; marcu.anca@umft.ro (A.M.); cmarian@umft.ro (C.M.); paula.muntean@umft.ro (P.D.C.)

**Keywords:** cortisol, total antioxidant capacity, saliva, stress, dental students

## Abstract

*Background and Objectives*: Stress is a significant issue among dental students, with both psychological and physiological impacts affecting their academic performance. This cross-sectional study aimed to explore the relationship between academic stress and salivary biochemical markers, specifically cortisol and total antioxidant capacity (TAC), in third- and fifth-year dental students during the 2022/2023 academic year. *Materials and Methods*: This study included 44 participants from the Faculty of Dental Medicine at Victor Babes University, Romania. Saliva samples were collected during a low-stress period and prior to exams (high-stress period). *Results*: Cortisol and TAC levels were measured using ELISA and TAC assays, respectively, decreasing from an average of 3.69 (SD ± 1.49) before stress to 2.86 (SD ± 2.28) during high-stress periods *(p* < 0.05), while salivary cortisol levels showed a non-significant change from 23.69 (SD ± 35.6) ng/mL to 20.44 (SD ± 23.07) ng/mL; additionally, female participants exhibited a mean STAI score of 91.65 (SD ± 5.13) compared to 91.09 (SD ± 9.41) for males, indicating greater stress responses among females. *Conclusions*: The State-Trait Anxiety Inventory (STAI) scores confirmed elevated anxiety levels during exams. The findings suggest that academic stress negatively impacts TAC while triggering a moderate increase in salivary cortisol, underlining the need for stress management interventions in dental education.

## 1. Introduction

Stress is the body’s reaction to physical or psychological challenges that disturb homeostasis, leading to various cognitive, emotional, and behavioral changes [1]. While short-term stress can enhance immune function, prolonged stress negatively impacts the immune system and is linked to numerous physical and mental health issues, such as infectious diseases, diabetes, cardiovascular conditions, and certain types of cancer [2].

Academic stress has been found to manifest in symptoms such as anxiety, depression, emotional exhaustion, fatigue, gastrointestinal issues, and sleep disturbances [3]. Furthermore, it has been shown to negatively impact educational performance [4]. Numerous studies [5,6,7] have shown that dental students experience significant levels of stress and anxiety throughout their training period. The demanding nature of dentistry begins early, as dental students are required to master a broad range of knowledge and skills to succeed both in their studies and their future careers [4]. While data on the effect of stress on the academic performance of dental students are inconsistent, there is evidence suggesting that high levels of perceived stress can lead to psychological issues and emotional exhaustion [8]. This, in turn, may increase the risk of professional burnout and reduced productivity.

It has been noted that stress levels vary depending on the year of study [9]. Most previous research has shown that students enrolled in clinical courses experience higher levels of stress. However, there is no clear agreement on which stage of academic education is linked to the peak of psychological stress. Some studies [10,11] suggest that the transition from preclinical to clinical training is particularly stressful, as students face their first interactions with patients and the responsibility of performing necessary treatments. Conversely, other researchers have found that the final year, where students focus on refining practical skills, is the most stressful, likely due to anxiety about their future professional careers. Additionally, stress levels tend to fluctuate throughout the academic year, with a notable increase during exam periods [10]. Studies also suggest that monitoring changes in specific salivary biochemical markers could provide a non-invasive and stress-free method for assessing stress levels, potentially helping to prevent mental health issues [12,13]. Examining the relationship between salivary parameters and stress in dental students can offer important insights into how stress affects oral health and aid the creation of preventive strategies and interventions [14].

Cortisol, often known as the stress hormone, is released by the adrenal cortex in response to stress. Measuring cortisol in saliva offers distinct advantages over traditional serum-based methods, as saliva collection is simple, is non-invasive, and does not induce stress, unlike blood sampling, which can elevate cortisol levels [6]. Advances in laboratory techniques have improved the sensitivity and specificity of salivary hormone measurements. In conditions where cortisol binding is altered, salivary biomarkers provide a more accurate reflection of adrenal function than serum cortisol. Furthermore, salivary cortisone has proven to be a more reliable indicator of serum cortisol, particularly in cases of low serum cortisol or during hydrocortisone therapy, where contamination may distort salivary cortisol results [15]. Cortisol is released in a circadian rhythm, beginning to rise between 2:00 and 4:00 a.m., peaking shortly after waking (the cortisol awakening response, or CAR), and gradually declining throughout the day, reaching its lowest point around midnight. To accurately assess cortisol levels, multiple samples must be collected over a 24 h period, which can be carried out using serum, saliva, or urine, each with its own advantages and disadvantages. Cortisol levels increase in response to physical or emotional stress and are disrupted by shift work and sleep deprivation. Salivary cortisol measurement is advantageous due to its non-invasive nature, whereas blood sampling can induce stress and potentially elevate cortisol levels artificially [15].

Several commercial kits are available for saliva collection, offering a convenient method for measuring cortisol levels, which remain stable in saliva. Samples can be stored at room temperature for a few days or refrigerated and frozen for longer periods, with stability maintained for up to three months at 5 °C and at least one year at −20 °C or −80 °C. However, long-term storage at room temperature is not recommended due to a gradual decrease in cortisol concentration over time. Importantly, saliva collection requires precautions to avoid contamination by blood or ingested substances, making it unsuitable for individuals with oral lesions, mouth ulcers, or poor oral hygiene [15]. Salivary cortisol levels are unaffected by saliva flow rate and accurately reflect the biologically active, unbound fraction of cortisol in plasma and serum, with only a 1–2 min delay between changes in plasma and saliva cortisol levels [1,16]. While freezing and thawing cycles do not affect cortisol levels, food intake within 90 min prior to sampling can alter measurements, as can certain medications like glucocorticoids and antidepressants, as well as conditions affecting the hypothalamic–pituitary–adrenal axis [17].

Oxidative stress arises from an imbalance between the production of free radicals and reactive oxygen species (ROS) and the body’s antioxidant defenses, potentially damaging lipids, proteins, and nucleic acids. This can result from excessive ROS production or reduced antioxidant levels, leading to cellular damage. Antioxidants, even in small amounts, can prevent or delay oxidation. Since the effectiveness of antioxidants varies, total antioxidant capacity (TAC) is often measured. Saliva and urine, being easier to collect, are frequently used to assess TAC, with lower salivary TAC indicating oxidative stress. Psychological factors, such as academic stress, have been linked to oxidative stress, which may reduce TAC and impair overall health. Academic stress, particularly among medical students, can lead to increased oxidative stress, negatively affecting cognitive function and exam performance [18]. TAC represents the combined activity of all antioxidants present in biological fluids. Many studies have suggested that oxidative stress plays a role in the development of various disorders, including cancer, cardiovascular conditions (such as atherosclerosis), diabetes mellitus, neurodegenerative diseases (like Alzheimer’s, Parkinson’s, and multiple sclerosis), rheumatoid arthritis, respiratory conditions (such as asthma), and the aging process [19,20].

The aim of this study was to explore potential associations between changes in specific biochemical markers in saliva (such as cortisol and total antioxidant capacity) and varying stress levels experienced by dental students throughout the academic year.

## 2. Materials and Methods

### 2.1. Study Participants

This cross-sectional study was carried out during the 2022/2023 academic year. The study was conducted between December 2022 and February 2023 at a single location, the Translational and Experimental Clinical Research Centre in Oral Health within the Clinic of Preventive, Community Dentistry and Oral Health, University of Medicine and Pharmacy “Victor Babes”, Timisoara, Romania. The participants, all students attending the Faculty of Dental Medicine, specifically third-year and fifth-year students, were sampled at two different time points: first, before the start of a course during the semester, and second, prior to an exam during the exam period under stressful conditions. Third-year and fifth-year students were selected for this study due to the distinct stress profiles associated with these academic stages: the third year marks a critical phase where students must pass all exams to progress to the next level, adding substantial academic pressure, while the fifth year presents intensive theoretical and clinical demands as students prepare for final evaluations, creating a unique set of stressors at this stage.

This study complied with the ethical guidelines set forth in the World Medical Association’s Declaration of Helsinki (1964). Approval was granted by the Ethical Committee of the University of Medicine and Pharmacy “Victor Babes”, Timisoara, Romania (no. 34/2018). Participation was entirely voluntary, and all participants provided informed consent by reading and signing the consent form.

Before this study began, its objectives were clearly explained to the dental students. Data were collected using a self-administered questionnaire, which was adapted from a previous study. Relevant participant information was recorded, including age, gender, general health status, medication use, and smoking habits (if applicable).

The inclusion criteria required participants to be in good general health, free from systemic diseases known to affect salivary TAC, such as diabetes, cardiovascular diseases, and chronic inflammatory conditions [21]. Participants were also required to have no oral mucosal lesions, have no diagnosed depressive or anxiety disorders, and not be pregnant. Exclusion criteria included the use of medications influencing oxidative balance (e.g., inhaled corticosteroids), high alcohol consumption, smoking, restrictive diets, or the use of dietary supplements. To ensure the integrity of the findings, participants were instructed to abstain from food, beverages (except water), and oral hygiene practices for at least two hours prior to saliva collection. These criteria were designed to control for external factors that could confound the relationship between stress and salivary biomarkers.

### 2.2. Saliva Collection

To control for potential diurnal variations in cortisol levels, all exams and corresponding saliva collections were conducted in the morning hours, specifically between 8 and 10 AM, minimizing the impact of circadian fluctuations on salivary cortisol concentrations [22]. This study was conducted over a consistent four-week exam period within the same academic term to reduce the influence of seasonal variations on salivary markers.

Unstimulated whole saliva was collected from study participants using the draining method, chosen for its simplicity and high acceptability. Participants were instructed to avoid food, beverages (except water), oral hygiene procedures, and smoking for specific periods before collection to standardize conditions and minimize contamination. Specifically, food and beverages were avoided for at least two hours, while smokers abstained for at least one hour prior to sampling. Additionally, participants refrained from taking any medications for at least eight hours before collection to avoid potential effects on salivary secretion.

During the collection, participants were seated in a relaxed position with their head slightly tilted downward, minimizing facial and lip movements. Saliva was allowed to drain passively into sterile containers for a period of 10 min without swallowing. To maintain the integrity of the samples, they were immediately stored at 4 °C in an ice container and transported to the laboratory within one hour for further processing. Consistent temperature control was critical to prevent biomarker degradation and ensure reliable readings for cortisol and TAC levels.

While this study focused on single-time-point cortisol measurements in the morning, we acknowledge that assessing cortisol levels at multiple time points during the day would better capture potential abnormalities in circadian rhythms. However, logistical constraints and participant compliance limited this approach. This limitation is discussed in the manuscript and highlighted as an area for future research.

### 2.3. Cortisol Level Determination

Biochemical analysis of the saliva was performed at the Department of Biochemistry and Pharmacology, Victor Babeş University of Medicine and Pharmacy. For the determination of cortisol levels in saliva, the Elabscience^®^ QuicKey Pro Human Cortisol ELISA Kit (Houston, TX, USA) was utilized. Saliva samples were collected and centrifuged for 10 min at 4000× *g* at a temperature of 2–9 °C to remove particulates, and the supernatant was used for analysis. The kit is based on the Competitive-ELISA principle, where samples or standards compete with pre-coated cortisol on the microplate for binding to a horseradish peroxidase (HRP)-conjugated antibody specific to cortisol. After incubation and washing steps, a substrate solution was added, and the reaction was stopped with a stop solution. The optical density (OD) was measured at 450 nm, and cortisol concentrations were determined by comparing the ODs of the samples to a standard curve prepared from serial dilutions of cortisol standards (ranging from 6.25 to 400 ng/mL). All assays were performed in duplicate to ensure accuracy, and intra- and inter-assay variability was maintained below 10% throughout the procedure.

### 2.4. TAC Determination

To maintain the integrity of the samples, they were kept at a constant temperature of 4 °C and transported to the laboratory for total antioxidant capacity (TAC) measurement within one hour of collection. TAC was assessed using a commercially available assay kit (ab65329, Abcam, Cambridge, UK). This kit operates by reducing Cu^2+^ to Cu^+^, with results expressed as Trolox equivalents (a well-known antioxidant standard). The saliva samples were centrifuged at 2500× *g* for 10 min at 4 °C to eliminate debris and impurities, after which TAC was measured following the manufacturer’s protocol using a GloMax^®^ Discover Microplate Reader (Promega, Madison, WI, USA). The TAC calculation was as follows:

*Sample Total Antioxidant Capacity* (TAC) = (*Ts*/*Sv*) × *D*

where *Ts* = TAC amount in the sample well calculated from standard curve (nmol), *Sv* = sample volume added in the sample well (µL), and *D* = sample dilution factor.

### 2.5. Assessment of Stress Level

To assess stress levels, the students completed the State-Trait Anxiety Inventory (STAI), a self-assessment questionnaire originally designed for adults but also suitable for high school students and individuals with psychiatric or physical disorders. The STAI consists of two parts: the State Anxiety Inventory (STAI-S) and the Trait Anxiety Inventory (STAI-T). The STAI-S measures temporary emotional reactions to specific situations, where anxiety levels vary depending on perceived threats. Participants report how they feel at a particular moment. The STAI-T measures general anxiety tendencies, reflecting how individuals typically feel in neutral situations. Both scales are scored on a 4-point scale, with total scores ranging from 20 to 80, classified as low (20–37), moderate (38–44), or high anxiety (45–80) [23,24].

### 2.6. Statistical Analysis

The statistical analysis was carried out using SPSS version 23 (IBM, Chicago, IL, USA). Descriptive statistics, including the calculation of mean and standard deviation, were performed for salivary cortisol levels and total antioxidant capacity (TAC). Given that we assessed the same participants under different conditions (low-stress vs. high-stress periods), a Wilcoxon signed-rank test was employed to compare group means over time, ensuring a more accurate analysis of changes in salivary biomarkers within the cohort. To ensure the suitability of parametric tests, we assessed the normality of data distributions using the Shapiro–Wilk test and visual inspection methods, such as Q-Q plots. This verification allowed us to confirm the appropriateness of parametric analyses or to consider non-parametric alternatives as needed. Additionally, Spearman Rho’s correlation coefficient was applied to assess the relationship between stressed markers (cortisol level, TAC and STAI) and mean age, gender and pathology frequency. A significance level of *p*-value < 0.05 was considered for all statistical analyses.

## 3. Results

A total of 44 students, consisting of 23 females (48.9%) and 21(44.7%), men took part in this study after providing written consent. The average age of participants is *M* = 24.51 (SD ± 5.47). Additionally, the majority reported being healthy, with 80.9% (*N* = 38) stating that they do not suffer from acute or chronic illnesses, while 12.8% (*N* = 6) indicated having such conditions (Table 1). Participants reported health conditions. Participants with chronic illnesses were retained in this study only if their conditions did not influence salivary total antioxidant capacity (TAC). Additionally, smokers and individuals with known respiratory pathologies were excluded to ensure that the oxidative stress markers and biomarker levels accurately reflected academic stress rather than external health factors.

Table 2 shows the descriptives for all the markers studied in both situations (stressed and non-stressed). As we can see, there were a total of 44 participants who completed both stages. For the cortisol marker, the average mean before the stressful situation was *M* = 23.69 (SD ± 35.6, while the average mean for cortisol concentration after the stressful situation was *M* = 20.44 (SD ± 23.07). For the TAC marker, we registered an average mean of *M* = 3.69 (SD ± 1.49) before the stressful situation, whilst after the stressful situation, we recorded an average mean of *M* = 2.86 (SD ± 2.28). Regarding the mean score for the STAI questionnaire, we recorded a value of *M* = 100.70 (SD ± 20.78) before the stressful situation and a mean value of *M* = 91.86 (SD ± 7.40) after the stressful situation.

The analysis of the distribution of TAC levels, cortisol concentration, and State-Trait Anxiety Inventory (STAI) scores under stressed and non-stressed conditions revealed significant variations. Statistical tests assessing the normality of these variables indicated notable deviations from a normal distribution for TAC levels under stress, suggesting non-normality. Cortisol concentrations in the non-stressed condition also showed substantial deviations from normality, whereas STAI scores were closer to a normal distribution in both conditions, with a more pronounced trend toward normality under stress. These findings highlight the need for non-parametric analyses for certain variables.

### 3.1. Costisol Levels

Table 3 presents a comparison of the mean salivary cortisol levels at two different time points for the participants. For this analysis, we used the Wilcoxon signed-rank test to compare the pre-test and post-test values of the paired variables. Regarding the comparison between cortisol in a non-stressed state and cortisol in a stressed state, we did not find a significant result, *Z*(44) = −0.15, *p* > 0.05 (*p* = 0.87) We can safely say that for these sample, there were not any significant differences between cortisol values.

**Figure 1:**
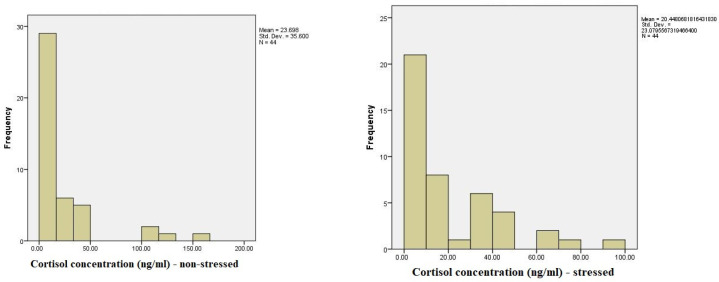
A comparison of salivary cortisol levels during low-stress and high-stress periods.

The distribution of cortisol levels in non-stressed situations shows a wide range of values, ranging from a minimum of 0.834 ng/mL to a maximum of 163.220 ng/mL. The majority of participants had cortisol levels clustered at lower concentrations, with 50% of the sample having concentrations below 10.017 ng/mL. Less frequent but notable higher concentrations were observed, with 3.6% of the sample showing cortisol levels above 105 ng/mL and 1.9% reaching the highest concentration of 163.220 ng/mL. This suggests variability in the baseline cortisol levels among participants, with most falling in the lower-to-moderate range, while a few individuals exhibited elevated levels (Figure 1).

The cortisol concentration (ng/mL) under stress shows a wide distribution of cortisol levels, with 25.5% of participants having levels that fall within the normal range of cortisol concentrations. The remaining participants exhibited more variable concentrations, ranging from as low as 0.252 ng/mL to as high as 98.838 ng/mL. A small number of participants (3.6%) had values labeled as “out of range”. The cumulative data reveal that most participants (50.9%) had cortisol concentrations below 20 ng/mL, while higher levels were progressively less frequent. Notably, several participants exhibited exceptionally elevated cortisol levels, with 1.8% of the sample showing concentrations above 70 ng/mL and the highest recorded value reaching 98.838 ng/mL. These results indicate a broad variability in the cortisol response to stress, with most participants exhibiting moderate increases, while a smaller subset displayed significantly elevated stress-related cortisol levels.

### 3.2. TAC Levels

**Figure 2:**
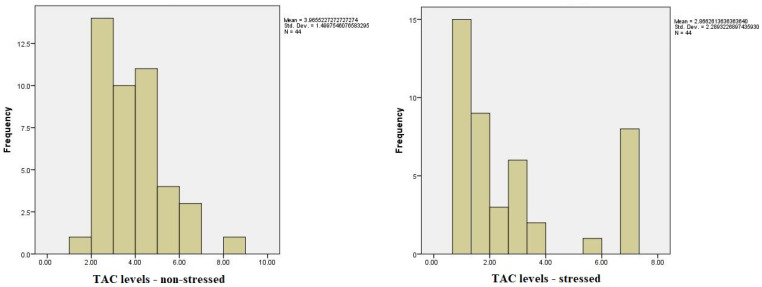
Total antioxidant capacity (TAC) in saliva under low- and high-stress conditions.

Regarding the comparison between the values of TAC (1) pre-test and (2) post-test, we did find significant differences using the Wilcoxon signed-rank test, *Z*(44) = −2.59, *p* < 0.05 (*p* = 0.010). The mean for TAC 1 was *M* = 3.96, *SD* = 1.49, whilst the mean for TAC 2 was *M* = 2.86, *SD* = 2.28; based on these results, we can say that the value of TAC 1 was higher before the stressful situation compared to the mean value after the stressful situation (Figure 2). Below, you can see a table showcasing and comparing the ranks between the values (Table 4).

### 3.3. STAI Levels

**Figure 3:**
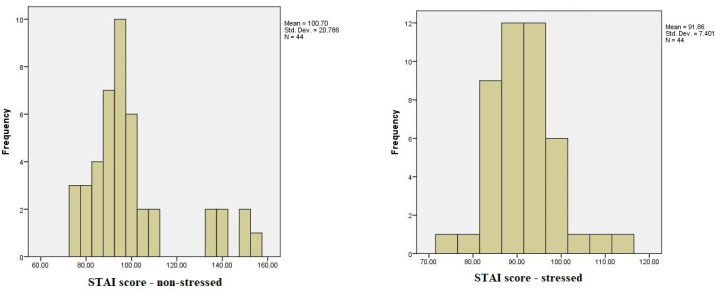
A comparison of STAI scores during low-stress and high-stress periods among dental students.

The results of the STAI questionnaire, which assesses both state and trait anxiety, revealed notable differences between the stress-free period and the period preceding exams. In the non-stressed state, overall STAI scores ranged from a minimum of 51 to a maximum of 148, with the majority of scores clustered around 84. In contrast, during the high-stress period before exams, STAI scores ranged from 46 to 132, reflecting an increase in anxiety levels. Using the STAI rating scale, we categorized anxiety into low (20–37), moderate (38–44), and high (45–80) levels for both state and trait anxiety. Under stress-free conditions, the average STAI-state and STAI-trait scores suggested a predominance of moderate anxiety, with fewer participants in the high-anxiety category. However, as exam stress increased, a shift towards higher STAI scores was observed, indicating an escalation in anxiety levels across participants (Figure 3).

Regarding the comparison between the values of STAI (*N*) pre-test and (*S*) post-test, we did find significative differences using the Wilcoxon signed-rank test, *Z*(44) = −2.40, *p* < 0.05 (*p* = 0.016). The mean for STAI (*N*) was *M* = 100.70, *SD* = 20.78, whilst the mean for STAI S was *M* = 91.86, *SD* = 7.40; based on these results, we can say that the mean score of STAI was higher before the stressful situation compared to the mean value after the stressful situation. Below, you can see a table showcasing and comparing the ranks between the values (Table 5).

To examine whether there are significant differences between genders regarding the independent variables studied under stress conditions, the Kruskal–Wallis test was performed. For this sample, the results indicate that there were no significant (*p* = 0.962, meaning that we cannot reject the null hypothesis) difference between sexes were found while looking at the descriptive data there were some differences regarding the mean (Table 6).

Table 6 presents the descriptive statistics comparing the concentration of cortisol (ng/mL), TAC 2, and STAI-S scores between male and female participants under stressed conditions. In terms of cortisol concentration, females had a slightly higher mean (*M* = 22.93, SD ± 25.32) than males (*M* = 17.72, SD ± 20.60), while for TAC stress levels, males showed a higher mean (*M* = 3.10, SD ± 2.42) compared to females (*M* = 2.64, SD ± 2.18). For the STAI stress scores, females had a marginally higher mean (*M* = 91.65, SD ± 5.13) compared to males (*M* = 91.09, SD ± 9.41). Although the observed differences in these measures were small, they offer insights into the variation in stress responses between males and females. These findings provide a nuanced understanding of how cortisol levels, antioxidant capacity, and anxiety responses may vary across genders in the context of stress.

To test whether we had a significant correlation between stressed markers (cortisol level, TAC and STAI) and mean age, gender, and pathology frequency, Spearman’s Rho correlation coefficient was used, and a significance level of *p*-value < 0.05 was considered for all statistical analyses. There was a significant positive correlation regarding the frequency of acute/chronic diseases and the gender of the participants, in the sense that women reported more acute/chronic diseases than men. For this, the non-parametric Spearman rho correlation Rho (44) = 0.38, *p* < 0.05 (*p* = 0.011) was used. The results have also shown that there is a significant negative correlation between the average TAC stress and the frequency of acute/chronic diseases present in patients, so that as the level of TAC stress increases, the frequency of acute/chronic diseases of the participants decreases Rho (44) = −0.30, *p* < 0.05 (*p* = 0.041) (Table 7).

## 4. Discussion

This study examined salivary cortisol and total antioxidant capacity (TAC) as biomarkers for academic stress among dental students, revealing a significant decrease in TAC during high-stress periods, while cortisol showed slight, non-significant variability. These findings underscore the physiological impact of academic stress, particularly through oxidative stress markers, and align with previous studies suggesting that stress-related antioxidant depletion may reflect the body’s adaptive response to sustained psychological pressure [25,26].

Although our study observed minimal cortisol variability, studies like Myint et al. (2021) [5] indicate significant increases during exam periods, suggesting that cortisol’s role in stress response may vary depending on personal stress perception and baseline levels [5,7]. Furthermore, the slight variability aligns with evidence that short-term academic stressors may not always elicit significant endocrine responses, particularly in healthy individuals [15,27].

While cortisol may not always show significant variation, the notable reduction in TAC suggests that oxidative stress markers may be more directly impacted during intense academic stress periods.

The significant TAC reduction in our study aligns with findings by Sivoňová et al. (2004) [26], who observed decreased antioxidant levels in students during exam periods. This decrease likely represents an adaptive response to oxidative stress, wherein antioxidant reserves are utilized to counteract reactive oxygen species (ROS) generated under psychological stress [26,28]. Similarly, Safabakhsh et al. (2022) demonstrated that salivary biomarkers, including total antioxidant capacity, are sensitive indicators of systemic and environmental factors, as evidenced by significant differences between obese and normal-weight individuals [29].

In addition to its impact on TAC and cortisol levels, stress perception may also vary significantly by gender, a noteworthy finding in our context.

Gender differences in stress perception were noted, with female participants generally exhibiting slightly higher stress levels, consistent with research by Al-Saleh et al. (2010) [30] and Hayes et al. (2017) [12], who attribute these variations to hormonal influences and stress response modulation. These findings suggest that stress management interventions might benefit from a gender-sensitive approach in educational settings.

These results underscore the importance of integrating stress-management programs, such as mindfulness and resilience training, within dental curricula. Studies show that these techniques can mitigate both physiological and psychological stress markers, improving student well-being and academic performance [13,31].

Prolonged stress, psychological distress, and/or emotional exhaustion can eventually result in professional burnout. This is particularly concerning when it occurs early in the educational phase. Stress, along with anxiety and depression linked to stress, is increasingly documented among medical and dental students in the literature [10,32]. Previous studies [5,7] have highlighted this trend. Healthcare education is both challenging and demanding, and the stress that it generates can negatively impact students’ academic performance, as well as their mental and physical well-being. These studies have also shown that perceived stress is connected to a reduced quality of life and early mortality [33]. Dental education, in particular, is considered one of the most stressful learning environments. It is both challenging and demanding, as it requires students to develop a wide range of skills, including academic knowledge, clinical abilities, and essential communication skills [4,34,35]. Examination stress induces particular physiological reactions in a student’s body that are adaptive and lead to changes in hormonal equilibrium [36].

It is crucial to acknowledge that, beyond academic stress, several lifestyle factors, such as sleep quality, dietary habits, and physical activity, may influence salivary cortisol and TAC levels. For instance, cortisol secretion follows a circadian rhythm, peaking in the morning and gradually decreasing throughout the day. However, disruptions in sleep patterns, commonly experienced during exam periods, could alter this rhythm and result in increased cortisol levels throughout the day. Similarly, total antioxidant capacity (TAC) can be significantly affected by diet, particularly the intake of antioxidant-rich foods. The depletion of antioxidants during periods of intense psychological stress could reflect both the body’s physiological response to stress and the impact of suboptimal dietary habits [31,37]. Future studies should consider controlling for these lifestyle factors to isolate the direct effects of academic stress on salivary markers [1,38].

The relationship between self-reported stress, salivary cortisol levels, and total antioxidant capacity levels (TAC) was examined in this study among a group of dental students. This was carried out using a self-administered stress questionnaire and by measuring the participants’ salivary cortisol levels and TAC levels.

This study evaluated the changes in salivary TAC and cortisol during periods of low and high academic stress in dental students. Salivary TAC decreased during high-stress academic periods, such as exams, compared to low-stress periods, suggesting that exam-related academic stress affects salivary TAC, though only for a short duration (during exam season) [1,38]. This reduction may be viewed as an actively protective response to inflammatory tissue changes, involving an increase in oxidative processes and a deficiency of antioxidants to neutralize the excess reactive oxygen species [33]. This finding aligns with Pani et al., who also observed a significant drop in salivary TAC during academic exams. It is important to note that stress levels may vary across different academic fields [28]. Akbari et al. found that 52% of dental students experienced abnormal stress levels throughout their education, with academic stress having a notably greater impact on their personal stress levels compared to other factors [18]. Sivoňová et al. [26] conducted a study evaluating oxidative stress in university students during exams. Their research, which involved 15 healthy medical students in both non-stressed and stressed conditions, demonstrated a reduction in plasma antioxidant levels during periods of stress. Similarly, a study by Eskiocak et al. produced comparable findings in seminal plasma, examining 34 medical students during both stressful and non-stressful periods [33].

The precise mechanisms through which oxidative stress markers function is still unclear [39]. A decline in antioxidant capacity could result from the exhaustion of antioxidants caused by oxidative stress. One potential explanation for the elevated oxidative stress observed during psychological stress might be the increased levels of catecholamines in the blood, which are known to generate reactive oxygen species [36].

Salivary cortisol levels can be measured in different units, such as ng/mL, µg/dL, and nmol/L, with conversions carried out using the following formulas (IBL International, 2009): cortisol (ng/mL) × 2.76 = nmol/L and cortisol (µg/dL) × 27.6 = nmol/L. The average salivary cortisol levels in healthy individuals are typically 0.20–1.41 µg/dL (5.52–28.92 nmol/L) in the morning and 0.04–0.41 µg/dL (1.10–11.32 nmol/L) in the afternoon. Cortisol secretion significantly increases during acute stress, with the amount of cortisol released correlating with stress intensity. Upon exposure to stress, adrenocorticotropic hormone (ACTH) levels rise within the first 5 min, while cortisol levels peak 10–30 min later. The transfer of cortisol from blood to saliva occurs quickly, within 2–3 min [27]. In interpreting cortisol concentrations, in our study, we referenced established norms from the literature, which typically define morning salivary cortisol levels in a low-stress context as ranging from approximately 0.20 to 1.41 µg/dL (5.52–28.92 nmol/L). Values falling outside this range in our study were noted as potential indicators of atypical stress responses, providing a basis for understanding individual variations in cortisol levels.

Krzysztol noted increased cortisol levels in reaction to academic and laboratory stressors [40]. Conversely, Pradhan et al. [41] observed reduced cortisol levels during exam-related stress, while Larson et al. [42] reported no notable changes in plasma cortisol levels before, during, or after the exam period [36].

In our study, female students scored higher than their male counterparts. These results align with those of Al-Saleh et al. [30] and Hayes et al. [12], which may be explained by psychological differences between genders. Females tend to express their emotions and experiences more openly than males. However, this perspective contrasts with the findings of Pani et al. [25], who reported higher salivary cortisol levels in males compared to females. These differences could be further explained by variations in coping mechanisms and hormonal regulation. Estrogen and progesterone have been shown to modulate the stress response in women, potentially leading to greater emotional sensitivity, while testosterone may contribute to the heightened cortisol response observed in men. Addressing these gender-based differences in stress management programs could enhance the effectiveness of interventions aimed at reducing both the psychological and physiological impacts of academic stress [1,43].

It is important to recognize certain limitations of the present study. A key limitation of this study is the small sample size, which may impact the statistical power and limit the generalizability of the findings. Another limitation of this study is that it was conducted at a single center. Future research should aim to include a larger and more diverse sample across multiple institutions involving students from multiple dental colleges to strengthen the reliability and applicability of these results to a broader population of dental students. Another limitation it that the inclusion of volunteers in this study may have introduced selection bias. Instead of comparing stress and other factors across different educational groups, this study focused on individuals with a specific educational background, namely dentistry. Since various educational systems present unique challenges and stress markers that can affect the neuroendocrine system, these findings cannot be generalized to other populations. While the participants, as dental students, are generally knowledgeable about and adherent to proper oral hygiene practices, which likely reduces the prevalence of periodontal disease and active caries, this study did not include specific dental or periodontal examinations to verify oral health status. Future research should incorporate a preliminary dental health assessment to exclude any underlying oral conditions that may influence salivary biomarkers and oxidative stress levels. Additionally, the results cannot be applied to individuals from diverse socioeconomic and occupational backgrounds. Future studies could explore the relationship between different educational backgrounds, stress, and changes in cortisol levels and TAC at various stages. Another limitation of this study is the fact that we did not account for the socioeconomic statuses (SESs) of participants, which can affect stress levels and oxidative status. Research indicates that lower SES is associated with higher allostatic load, reflecting increased chronic stress and its physiological consequences. Secondly, while we excluded individuals with known acute or chronic systemic conditions, undiagnosed or subclinical conditions may have influenced the results. Systemic health issues can alter oxidative stress markers, potentially confounding our findings. Future research should incorporate comprehensive assessments of participants’ socioeconomic backgrounds and conduct thorough health screenings to control for these variables. Despite these limitations, the current study offers valuable insights into salivary cortisol levels and TAC levels among young university students.

The present study highlights the importance of early intervention in managing academic stress, particularly among dental students, who are exposed to prolonged periods of both psychological and physical stress. Implementing stress reduction programs, such as mindfulness-based stress reduction (MBSR) techniques or relaxation exercises, could potentially mitigate the negative effects of stress on both cortisol levels and TAC. Given the association between elevated stress and the risk of burnout, these strategies could improve students’ overall well-being and academic performance. Furthermore, integrating nutritional counseling focused on antioxidant-rich diets might play a role in reducing oxidative stress and preserving overall health during high-stress periods [38].

Despite the insights provided by this study, certain limitations must be acknowledged. A significant limitation of this study is the exclusion of personal clinical and sociodemographic variables, which could have provided a more comprehensive understanding of the factors influencing salivary biomarkers under stress. Additionally, the absence of multiple regression analysis limits the ability to explore complex relationships between variables, such as the interplay between cortisol levels, total antioxidant capacity, and anxiety scores. Future research should aim to incorporate these factors to better understand the multifactorial nature of stress responses in dental students.

## 5. Conclusions

This study underscores the physiological impact of academic stress on dental students, particularly through significant reductions in salivary total antioxidant capacity (TAC) during high-stress periods, suggesting heightened oxidative stress responses. While cortisol levels exhibited minor changes, the overall pattern supports the role of oxidative stress as a primary physiological marker under academic strain. These findings reveal gender-specific stress responses and indicate the need for effective stress management interventions in dental education. Future studies should consider a comprehensive approach, examining lifestyle factors, socioeconomic status, and broader educational settings to develop targeted health strategies that support student resilience and prevent burnout.

## Figures and Tables

**Table 1:** Descriptive statistics for age, gender, pathology.

Variable		N (%)
Gender	Male	21 (44.7%)
	Female	23 (48.9%)
Age	Average age (M ± SD)	24.516 ± 5.4764
General conditions (acute/chronic)	Yes	6 (12.8%)
	No	38 (80.9%)

**Table 2:** Descriptive statistics for concentrations of the markers in stress versus non-stress states.

	N	Mean	Std. Deviation	Std. Error Mean
Non-stressed cortisol concentration (ng/mL)	44	23.69	35.600	5.366
Stressed cortisol concentration (ng/mL)	44	20.44	23.07	3.47
TAC N	44	3.69	1.49	0.22
TAC S	44	2.86	2.28	0.34
STAI N	44	100.70	20.78	3.13
STAI S	44	91.86	7.40	1.11

**Table 3:** Salivary cortisol levels at various time points for the participating students (*N* = 44).

		N	Z	Mean Rank	Sum of Ranks
Non-stressed cortisol concentration (ng/mL)	Negative Ranks	23 a		22.09	508.00
Stressed cortisol concentration (ng/mL)	Positive Ranks	21 b		22.95	482.00
	Ties	0 c	−0.152 (0.87)		
	Total	44			

a. Stressed cortisol concentration (ng/mL) < non-stressed cortisol concentration (ng/mL). b. Stressed cortisol concentration (ng/mL) > non-stressed cortisol concentration (ng/mL). c. Stressed cortisol concentration (ng/mL) = non-stressed cortisol concentration (ng/mL).

**Table 4:** TAC levels at various time points for the participating students (*N* = 44).

		N	Z	Mean Rank	Sum of Ranks
TAC Stressed—TAC Non-stressed	Negative Ranks	34 a		21.09	717.00
	Positive Ranks	10 b		27.30	273.00
	Ties	0 c	−2.59 (0.01)		
	Total	44			

a. TAC S < TAC N. b. TAC S > TAC N. c. TAC S = TAC N.

**Table 5:** STAI levels at various time points for the participating students (*N* = 44).

		N	Z	Mean Rank	Sum of Ranks
STAI_S—STAI_N	Negative Ranks	29 a		22.21	644.00
	Positive Ranks	13 b		19.92	259.00
	Ties	2 c	−2.40 (0.01)		
	Total	44			

a. STAI_S < STAI_N. b. STAI_S > STAI_N. c. STAI_S = STAI_N.

**Table 6:** Group descriptive statistics for cortisol, TAC, and STAI-S by sex (stressed condition).

Variable	Sex (M/F)	N	Mean	Std. Deviation	Std. Error Mean
Stressed cortisol concentration (ng/mL)	M	21	17.72	20.60	4.49
	F	23	22.93	25.32	5.28
TAC-stress	M	21	3.10	2.42	0.52
	F	23	2.64	2.18	0.45
STAI-stress	M	21	91.09	9.41	2.05
	F	23	91.65	5.13	1.06

**Table 7:** Non-parametric correlation (*N* = 44).

			1	2	3	4	5	6
Spearman Rho	Age		-					
	Gender		−0.31	-				
	Pathology		−0.18	0.38 * (0.01)	-			
	Cortisol Stressed		0.04	0.07	−0.08	-		
	TAC S		0.23	−0.17	−0.30 * (0.04)	0.13	-	
	STAI S		0.13	0.02	−0.05	0.01	-0.01	-

Notes: *p* < 0.05 *.

## Data Availability

The data presented in this study are available on request from the corresponding author.
